# Is Alcohol Food?

**Published:** 1863-09

**Authors:** 


### IS ALCOHOL FOOD?

By the Editor of the British Medical Journal.

We possess no proof positive of the fact so very generally assumed, that alcohol is a food.

It may be most fairly suggested, until the contrary be shown, that alcohol acts by stimulation of the nervous system, by hastening and assisting the vital functions, the wear and tear of the body, the disintegration of the tissues, and the manufacture of lymph; and that by such disintegration of tissues the wasting body supplies itself with food during the period of sickness,—in fact, lives upon itself. The alcohol, we may argue, is the stimulant and promoter of the disintegration. It is indirectly the cause of the production of food, but assuredly not (*if such be its only action,*) food in itself. Until the contrary be shown, this, we affirm, is the only fair and legitimate conclusion to be deduced from the premises before us.

Now, if proof could be given that a person existing for some days solely upon an alcoholic-drink regimen gained in weight, or even did not lose in weight, then we should possess strong *prima facie* evidence that alcohol is a food. But when we find that the person who has been kept alive all these days by alcoholic drinks,—*i. e.*, by water *plus* alcohol,—has lost greatly in weight, has become emaciated, has used up his own flesh and blood in the preservation of his life,—in such case, we have assuredly no right to say more of the alcohol than this: that it acted a useful, or even it may be admitted, an essential part, in the struggle for life. We repeat it, we have no proof that, under such conditions, it did more than act as a stimulus.

Hence, then, as it seems to us, the clinical facts which some writers have produced as demonstrative of the food-nature of alcohol are, as such, worth absolutely nothing. The proof here must be rigid,—one of the scale and balance kind. Let us be told what the weight of the patients was *before* the experiment was commenced, and what *after*. Let us know how much water was swallowed with the alcohol; and be satisfied that nothing but diluted alcohols were taken while the experiments were going on,—that rigid abstinence from other things was positively maintained. The analysis of such facts would enable us to arrive at something positive on the subject.

We have no hesitation in saying, that to call alcohol food, in the present state of our knowledge of its effects, is an abuse of language. Those substances only can rightly be called food which are essential for the purposes of life; which form a part of the healthy body; which are capable (under the influence of the organic processes,) of being incorporated, wholly or in part, decomposed or undecomposed, with the body,—assimilated, as physiologists call it. We do not call tonics, which assist the primary digestion in the stomach, food; and if (which we say not,) it be true that alcohol is of use only as aiding, by stimulation, in the secondary digestion,—the disintegration of the tissues,—neither can we call alcohol food. Let it be clearly understood that we do not in this deny that alcohol is a food. What we affirm is, that we possess no particle of satisfactory and scientific evidence to show that it is such. Those who affirm it to be, should give us something like a tangible proof of the fact,—something beyond the mere vague surmises of their own opinions. Let them show that a body fed solely on alcoholic drinks for several days has gained, or at least not lost in weight; and they will have some facts upon which to found the assertion. But to say that an emaciated creature who rises from his bed of sickness, and has swallowed during his sickness large quantities of water and alcohol, is a living proof that alcohol is food, is manifestly an unfounded assumption. We have, as already stated, just as good, and in truth much better, grounds for saying that the alcohol acts solely by its power in assisting secondary digestion, in aiding the wasting of the body, the wear and tear of it, and thereby producing materials,—lymph,—for the support of the system at a time when disease has arrested the functions of primary digestion, &c.—*British Medical Journal.*

